# Construction of Chromosome Segment Substitution Lines and Inheritance of Seed-Pod Characteristics in Wild Soybean

**DOI:** 10.3389/fpls.2022.869455

**Published:** 2022-06-17

**Authors:** Haiyang Zheng, Lilong Hou, Jianguo Xie, Fubin Cao, Ruru Wei, Mingliang Yang, Zhaoming Qi, Rongsheng Zhu, Zhanguo Zhang, Dawei Xin, Candong Li, Chunyan Liu, Hongwei Jiang, Qingshan Chen

**Affiliations:** ^1^Northeast Agricultural University, Harbin, China; ^2^Jilin Academy of Agricultural Sciences, Soybean Research Institute, Changchun, China; ^3^Jiamusi Branch Institute, Heilongjiang Academy of Agricultural Sciences, Jiamusi, China

**Keywords:** CSSLs, high-density Bin-Map, QTLs, wild soybean, seed-pod characteristics

## Abstract

Genetic populations provide the basis for genetic and genomic research, and chromosome segment substitution lines (CSSLs) are a powerful tool for the fine mapping of quantitative traits, new gene mining, and marker-assisted breeding. In this study, 213 CSSLs were obtained by self-crossing, backcrossing, and marker-assisted selection between cultivated soybean (*Glycine max* [L.] Merr.) variety Suinong14 (SN14) and wild soybean (*Glycine soja* Sieb. et Zucc.) ZYD00006. The genomes of these 213 CSSLs were resequenced and 580,524 single-nucleotide polymorphism markers were obtained, which were divided into 3,780 bin markers. The seed-pod-related traits were analyzed by quantitative trait locus (QTL) mapping using CSSLs. A total of 170 QTLs were detected, and 32 QTLs were detected stably for more than 2 years. Through epistasis analysis, 955 pairs of epistasis QTLs related to seed-pod traits were obtained. Furthermore, the hundred-seed weight QTL was finely mapped to the region of 64.4 Kb on chromosome 12, and *Glyma.12G088900* was identified as a candidate gene. Taken together, a set of wild soybean CSSLs was constructed and upgraded by a resequencing technique. The seed-pod-related traits were studied by bin markers, and a candidate gene for the hundred-seed weight was finely mapped. Our results have revealed the CSSLs can be an effective tool for QTL mapping, epistatic effect analysis, and gene cloning.

## Introduction

Cultivated soybean (*Glycine max* [L.] Merr.) is an important food crop and oil crop in the world, and it is the most important source of plant protein (Nadeem et al., 2021). Therefore, the development of soybean industry is of great significance. Good variety is an important prerequisite for high yield and high yield of soybean. According to various traits of soybean, cultivating good varieties and improving yield and quality of soybean are the goals of breeding workers (Reinprecht et al., 2006). The emergence of molecular marker-assisted selection (MAS) and QTL mapping has significantly shortened the breeding time (Hasan et al., 2021). Some excellent loci and genes that are of great significance to breeding have been identified by populations, such as F_2_, BC_1_, DH, RIL, and IF_2_ (Mian et al., 1996; Song et al., 2005; Reinprecht et al., 2006; Alfred et al., 2012; Botero-Ramirez et al., 2020). The genetic composition of each member in the above population is diverse, which can provide abundant genetic variation information and calculate multiple genetic effects. However, the diversity of genetic background will also bring some interference to QTL mapping and affect its accuracy (Zhao et al., 2009; Qiao et al., 2016).

The CSSLs constructed based on backcrosses have similar genetic backgrounds, which can eliminate interference from genetic noise and improve the accuracy of QTL mapping (Ali et al., 2010; Balakrishnan et al., 2019). CSSLs are constructed by continuous backcross between recurrent parent and donor parent. The genetic background is clean and uniform, and only a few or a single donor genome introgressed fragments are different among the lines. CSSL population can accurately locate QTL loci for target traits. In addition, the process of backcrossing can introduce valuable genes into cultivated varieties from wild and distant species to broaden the genetic base of cultivated species. For more than 20 years, since Eshed and Zamir identified QTLs related to fruit solids, plant height, and green fruit weight in tomato (Alpert and Tanksley, 1996), CSSLs have received extensive attention. At present, more than 100 sets of CSSLs have been constructed in 20 crops. At the same time, a large number of QTLs were identified by these CSSLs, and fine mapping and gene mining of these QTLs was carried out (Tian et al., 2006; Gu et al., 2015; Wang et al., 2016b; Xin et al., 2016; Zhang et al., 2019).

As high-quality genetic populations, the above CSSLs can help to understand the genetic basis of crop traits and mining important candidate genes. At the same time, marker density has become the limiting factor in gene detection efficiency. Resequencing technology can provide high-density single-nucleotide polymorphism (SNP) markers. Employing SNP data to divide bin markers in the whole population is a feasible marker strategy that can detect QTLs more effectively than traditional methods. At present, it has been employed for fine mapping of QTLs for yield traits in rice, maize, and other crops (Fonceka et al., 2012; Chen et al., 2014; Gu et al., 2015; Wu et al., 2020).

Annual wild soybean is considered to be the wild ancestor of cultivated soybean (Broich and Palmer, 1980). In the process of domestication and improvement, pods, seeds, and other important yield traits have been under constant selection. At present, a large number of seed-pod trait QTLs have been detected. For example, Li et al. (2019) carried out QTL analysis and locus interaction effect analysis on the number of three-seed pods in a high-generation recombinant inbred line by a SLAF-seq high-density genetic map, and a total of 17 QTLs were detected. Han et al. (2020) performed additive QTL and epistatic QTL mining on the number of seeds, seed weight, number of pods, three-seed pods, and four-seed pods of recombinant inbred lines derived from the crossing of Hefeng 25 and Lmuri 28 by a molecular genetic linkage map, which was constructed by the Bin-Map mapping technique, and 27 related QTLs were obtained. However, only *Ln*, *GA20OX*, *SWEET10*, and *PP2C-1* were confirmed to be associated with seed-pod traits (Lu et al., 2016, 2017; Sayama et al., 2017; Wang et al., 2020). Furthermore, the allelic differences between wild and cultivated soybeans for these traits are largely unknown.

In this study, based on the original CSSLs, the genome was resequenced to QTL mapping of seed-pod traits. The purpose of this study was to broaden the genetic base of cultivated soybean, explore the genetic mechanisms of the formation of soybean pod traits, and mine the excellent genes of wild soybean by wild soybean resources. It is expected that the CSSLs will provide excellent breeding and genetic material and support for breeders and geneticists. Revealing the genetic mechanism of traits and mining important candidate genes could provide important theoretical support for the analysis of seed-pod trait formation in soybean.

## Materials and Methods

### Field Planting and Management of Soybean

The experimental materials used in this study included 213 CSSLs. The parents were SN14 (recurrent parent) and ZYD0006 (donor parent), constructed with the aid of simple sequence repeats (SSR) markers (Jiang et al., 2020). The materials of CSSLs consist of eight generations (6 BC_3_F_5_, 37 BC_3_F_6_, 61 BC_3_F_7_, 2 BC_4_F_4_, 12 BC_4_F_5_, 22 BC_4_F_6_, 13 BC_5_F_4_, and 60 BC_5_F_5_). During 2016–2020, the CSSLs were planted at Xiangyang Farm, Harbin, Heilongjiang Province (Harbin, latitude 45°45′N, longitude 126°38′E) in May every year. A randomized complete block design with three replicates was used for 5 years. The lines were grown in a one-row plot with 60 cm row spacing and 6 cm between plants. The rows were 5 m long, with approximately 80 plants per row. The germplasm resource population was planted in Xiangyang Farm, and the planting method is the same as CSSLs. To fine-map the HSW trait, a CSSL (R183) with small HSW was selected as male parent to cross with SN14. Then, the F_1_ plants were selfed in Hainan Experimental Station with the false hybrids removed. After selfing, 74 F_2:3_ and 144 RHL lines, which comprised the secondary population named R183-F_2:3_ and RHL, were harvested. Farmland management of all the above materials follows common agricultural practices.

### Soybean Phenotypic Measurement

In October every year, after the soybean reaches full maturity, five single plants with the same growth state were selected from each plant line to measure the pod-seed traits. After dried, measure the pod-seed traits in November of each year. Seed traits include seed length (SL), seed width (SW), hundred-seed weight (HSW), and seed-pod weight (SPW). Pod-related traits include the proportion of one-seed-pod-numbers (PoOSPN), the proportion of two-seed-pod-numbers (PoTSPN), the proportion of three-seed-pod-numbers (PoTHSPN), and the proportion of four-seed-pod-numbers (PoFSPN). Seed length is the sum of the longest distance parallel to the hilum of 10 seeds. Seed width is the sum of the longest distance perpendicular to the hilum of 10 seeds. The HSW is the weight of 100 seeds of uniform size. Seed-pod weight is the weight of all seeds and pod shells on the whole plant (Yang et al., 2017).

### Material Resequencing and Bin Marker Acquisition

Genomic DNA was extracted from young leaves of Suinong 14 (SN14), wild soybean ZYD00006, and CSSLs by a modified CTAB method (Wu et al., 2020). Then, the extracted genomic DNA was randomly interrupted by ultrasonic fragmentation, and the sequencing library was constructed by terminal repair, adding an a at 3′ end, adding a sequencing connector, purification, and PCR amplification. After passing a quality inspection, the library was sequenced with an Illumina HiSeqTM sequencing platform, and the sequence read length was 150 bp. By BWA and GATK software, a sequence was compared with the reference genomic *Glycine max Wm82.a2.v1* to identify the original SNP (Li and Durbin, 2009; McKenna et al., 2010; Schmutz et al., 2010), and high confidence SNPs were obtained according to the method of Guo et al. (2015).

A developed approach of sliding-window (Huang et al., 2009) was used for CSSLs genotyping with a group of 17 consecutive SNPs and a step size of 1. The position of a SNP that switched one genotype to another consecutive genotype was recorded as the recombination breakpoint. A bin map was constructed according to the recombination breakpoint information. All of the breakpoints along the same chromosome were sorted by physical position.

### Method of Date Analysis

Phenotypic data of CSSLs and secondary segregation population (F_2_, F_2:3_, and RHL) have been statistically analyzed by SPSS 26. For example, average value, maximum value, minimum value, etc. The best linear unbiased estimate (BLUE) can more effectively eliminate the deviation caused by the environment and improve the accuracy of QTL mapping. The BLUE was calculated by BLUE calculation option in the AOV module of the ICIMapping 4.2 software. The parameters used were each line of CSSLs as fixed factor, years and repetition as random factors.

### QTL Mapping of Pod-Seed Traits

Bin markers were used for QTL mapping of the seed-pod-related traits in CSSLs, and a likelihood ratio test based on stepwise regression model (RSTEP-LRT-ADD) with ICIMapping 4.2 software was used to determine QTLs. Parameters setting was “By condition number” = −1,000 (equivalent to deleting duplicate markers), “PIN” = 0.001 (PIN: the largest-value for entering variables in stepwise regression of residual phenotype on marker variables), and LOD value ≥2.5 (Wang et al., 2006, 2016a; Li et al., 2007, 2008). The linear model of the RSTEP-LRT method was as follows:


$$y_{i}=b_{0}+\overset{t}{\underset{j=1}{\sum }}b_{j}x_{ij}+e_{i.}$$

where *i* = 0 (for the recurrent parent), 1, 2, …, n, *b_0_* is the intercept, *b_j_* (*j* = 1, …, t) is the partial regression coefficient of phenotype on the *j*th marker, which represents QTL additive effect on each segment, *x_ij_* is the indicator variable for the *j*th marker in the *i*th CSSL, which is equal to −1 if the marker type is the same as in *P*_1_ (the recurrent parent) and 1 if the marker type is the same as in *P*_2_ (the donor parent), and *e_i_* is the random experimental error following a normal distribution.

DNA was extracted from the secondary segregated population by the CTAB method. SSR markers were used as molecular markers (the marker obtained from Soybase).^1^ After polymerase chain reaction (PCR) of Panaud, the genotype of secondary segregated population was then detected by 8% PAGE separation (Doyle and Doyle, 1990). Genetic map is constructed by MAP module in ICIMapping 4.2 software. QTL mapping employed the ICIM-ADD model with 1,000 permutations calculation of ICIMapping 4.2 software. And the threshold of ICIMapping for QTL detection was chose *p* ≤ 0.05. The epistasis of the seed-pod-related traits by the RSTEP-LRT-EPI model with PIN = 0.000001calculation of ICIMapping 4.2 software (Wang et al., 2007). And the threshold of ICIMapping for epistasis QTL detection were set chose LOD > 10.

### qRT-PCR Analysis of Candidate Genes

The seed of qRT-PCR material was sampled according to different developmental stages of soybean seeds. The Cotyledon Stage (COT, 10 days after flowering (DAF)) and Early Maturity Stage 1(EM1, 20 DAF) mainly carry out cotyledon development and endosperm assimilation. Then, the seed size and weight are increasing at Early Maturity Stage 2(EM2, 30 DAF) and Mid Seed Maturity (MM, 50 DAF).^2^ During the COT, EM1, EM2, and MM of seed in parents SN14, CSSL-R183, and wild soybean ZYD00006 (Severin et al., 2010; Collakova et al., 2013; Lu et al., 2016), soybean seed RNA was extracted by TRIzol (Invitrogen, 15596-026, Carlsbad, CA, United States). qRT-PCR was carried out on a Roche LightCycler 480 II fast real-time PCR system using SYBR qPCR Mix (Vazyme, Q711, Vazyme Biotech, Nanjing, China). *Actin* (*GLYM18G52780*) was used as the internal reference (Gu et al., 2017), and the relative amount of gene expression (target gene/actin) was calculated by the 2^ΔCT^ method. Premier 5.0^3^ was used to design qRT-PCR primer sequences for candidate genes.

### Haplotype Analysis

A total of 129 germplasm resources from China were used for haplotype analysis due to their rich genetic variation and large range of HSWs. The genomic sequences of the candidate genes were proposed on the Phytozome13 website,^4^ including the 5′UTR, upstream 2000 bp promoter region sequence, CDS, intron sequence, and 3′UTR sequence information. The sequence information for the candidate gene and the genomic sequencing information from the resequencing of 129 soybean germplasm resources were subjected to local BLAST analysis to obtain SNP information from the candidate genes in the germplasm resource population (unpublished). In this study, Dnasp5.0 software was used to analyze the haplotype distribution of candidate gene SNP sequences in germplasm resource populations, and excellent haplotypes (the number of cultivars belonging to the haplotype exceeding 5% of the total) were screened out.^5^ Using the Haps Format module in Haploview 4.2 software (Cambridge, Massachusetts, United States) to analyze the excellent haplotype sequence information from candidate genes, ANOVA of candidate gene haplotypes and phenotypes was performed by SPSS 26.0 software (Armonk, New York, USA), to determine the effect of each haplotype on phenotype. Plant cis-acting regulatory DNA elements (PLACE)^6^ and plant promoters and cis-acting regulatory elements (Plant Care)^7^ were used to query the function of plant promoter elements.

## Results

### Resequencing of CSSLs (Parents) and Division of Bin Markers

The sizes of the sequenced data from SN14, ZYD00006, and the 213 CSSLs were 30.08 Gb, 33.38 Gb, and 1410.31 Gb, respectively. The terminal reads of short pairs were mapped back to the Williams82 reference genome (Schmutz et al., 2010), and the results showed that the average alignment efficiency of SN14, ZYD00006, and the CSSLs was more than 90%. The average genome coverage depth and percentage of parents were more than 27× and 95% (covering at least 1×), respectively. The average genome depth and percentage of the CSSLs were more than 5.79× and 95.74% (covering at least 1×), respectively (Tables 1, 2). At the same time, 580,524 SNPs were identified between SN14 and ZYD00006, which indicated that there were genetic differences between cultivated soybean SN14 and wild soybean ZYD00006. These SNPs in the parents of the CSSLs also facilitate further fine mapping of QTLs.

**Table 1:** Chromosome coverage of parents and CSSLs.

ID	Clean reads	Mapped (%)	Properly_mapped (%)
Suinong14	111,279,380	98.29	92.33
ZYD00006	100,265,276	97.31	91.91
CSSLs (average)	44,174,311	98.73	93.45

Clean reads: The number of Clean Reads; Mapped (%): The percentage of the number of Clean Reads located to the reference genome to the number of all Clean Reads; Properly mapped (%): The percentage of paired-end sequencing sequences that are mapped to the reference genome and the distance from the sequenced fragments is in line with the length distribution; Offspring (average): The average value of CSSL-related indicators.

**Table 2:** The average coverage depth of parents and CSSLs.

ID	Ave depth	Cov_ratio_1X (%)	Cov_ratio_5X (%)	Cov_ratio_10X (%)
Suinong14	29	98.35	97.25	96.06
ZYD00006	27	98.92	96.92	94.57
CSSLs	5.79	95.74	63.2	15.29

Ave depth: The average coverage depth of the sample; Cov_ratio: The ratio of the number of bases whose coverage depth is at a given depth and above to the total number of bases in the reference genome.

**Figure 1:**
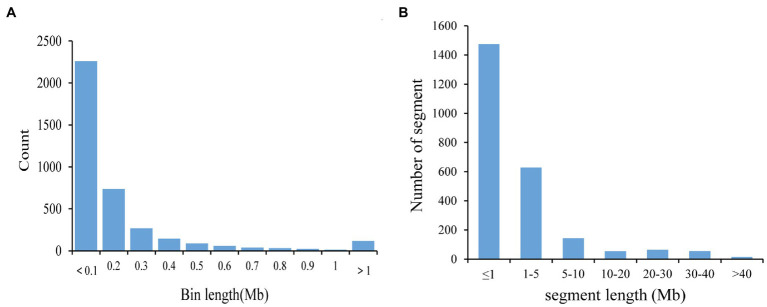
Bin and introgressed fragments of CSSLs. **(A)** Distribution of the physical lengths of the bins. **(B)** Frequency of introgressed fragments according to length.

The 580,524 SNP was divided into 3,780 bin markers. The range of bin marker length was 20 Kb–22.91 Mb, and the average marker length was 225.12 Kb. 79.26% of bin markers were less than 0.2 Mb in length, and 111 markers were more than 1 Mb in length (Figure 1A). There were 6 bin markers greater than 10 Mb, which were distributed on Chr. 04 (Bin2350), Chr. 08 (Bin4681), Chr. 09 (Bin4865), Chr. 12 (Bin6269), Chr. 18 (Bin9389), and Chr. 19 (Bin9688). At the same time, there were differences in the number and density of bin markers on 20 soybean chromosomes (Table 3). The highest and lowest bin marker densities were on chromosome 9 (2.33 markers/Mb) and chromosome 3 (5.50 markers/Mb), respectively. The average marker density was 4.04 markers/Mb, and the average distance between bin markers was 25.45 Kb. These CSSLs carried 2441 introgressed segments (including 459 introgressed segments with a single bin marker). The length of these introgressed segments ranged from 0.02 Mb to 51.74 Mb. Among the above introgressed segments, 60.43% of the introgressed segments were less than 1 Mb, and large segments greater than 10 Mb accounted for 7.87% (Figure 1B). The CSSLs many large introgressed fragments, so they need to be further expanded.

**Table 3:** The bin marker number and marker density across 20 chromosomes of the CSSLs.

Chromosome	Bin number	Average (Mb)	Maximum (Mb)	Minimum (Mb)	Marker/Mb
1	143	0.33	5.35	0.02	2.52
2	229	0.18	9.40	0.02	4.71
3	252	0.16	1.86	0.02	5.50
4	148	0.32	21.66	0.02	2.83
5	191	0.20	2.00	0.02	4.52
6	197	0.23	9.73	0.02	3.83
7	187	0.20	6.60	0.02	4.19
8	192	0.23	11.80	0.02	4.01
9	117	0.41	22.91	0.02	2.33
10	186	0.26	7.63	0.02	3.61
11	155	0.19	2.85	0.02	4.46
12	165	0.23	10.24	0.02	4.12
13	231	0.18	3.60	0.02	5.04
14	156	0.28	6.35	0.02	3.18
15	261	0.18	3.23	0.02	5.04
16	176	0.20	2.81	0.02	4.65
17	191	0.19	1.74	0.02	4.59
18	156	0.34	14.79	0.02	2.69
19	239	0.20	19.89	0.02	4.71
20	208	0.20	3.63	0.02	4.34
Average	189	0.24	8.40	0.02	4.04

### Phenotypic Variation and Statistical Analysis of CSSLs

**Figure 2:**
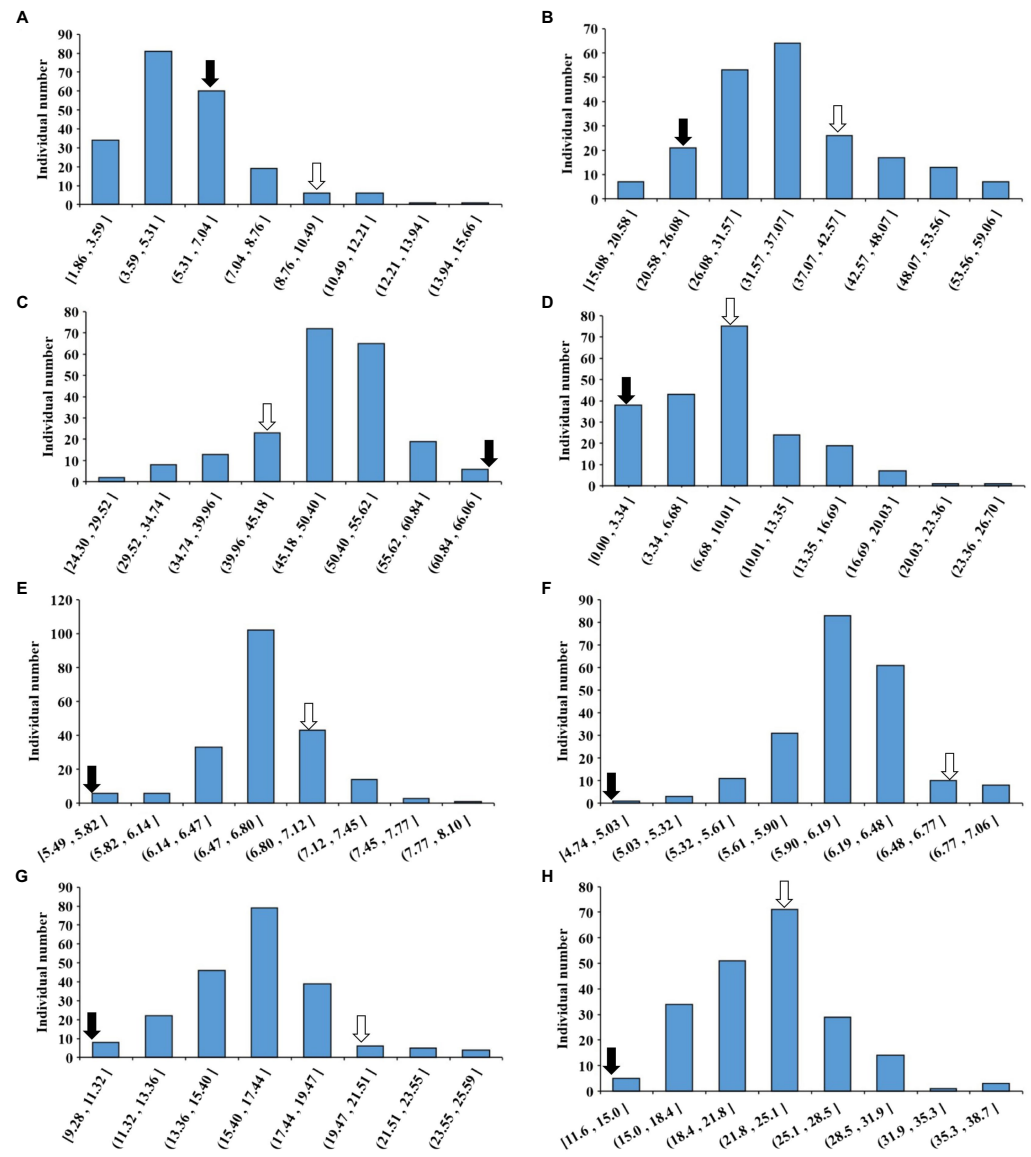
Phenotype of CSSLs seed-pod characteristics in BLUE. **(A)** The BLUE value of PoOSPN (%); **(B)** The BLUE value of PoTSPN (%); **(C)** The BLUE value of PoTHSPN (%); **(D)** The BLUE value of PoFSPN (%); **(E)** The BLUE value of SL (cm); **(F)** The BLUE value of SW (cm); **(G)** The BLUE value of HSW (g); **(H)** The BLUE value of SPW (g). ⇒: Suinong14, ➡: ZYD00006.

The phenotypic data of CSSLs from 2016 to 2020 were statistically analyzed and the BLUE was calculated. The maximum and minimum values of PoOSPN were 15.66 and 1.86%; the maximum and minimum values of PoTSPN were 59.06 and 15.08%; the maximum and minimum values of PoTHSPN were 66.06 and 24.29%; the maximum and minimum values of PoFSPN were 26.70% and 0; the maximum and minimum values of SL were 8.10 and 5.49 cm; the maximum and minimum values of SW were 7.06 and 4.73 cm; the maximum and minimum values of HSW were 25.69 and 9.83 g; and the maximum and minimum values of SPW were 38.68 and 11.62 g, respectively (Figure 2; Supplementary Table 1). The 5-year total broad heritability of each trait was 0.5014, 0.8462, 0.7286, 0.8054, 0.6474, 0.6626, 0.8821, and 0.4296, respectively (Supplementary Table 2). The above results showed that the phenotypic variation in the CSSLs was plentiful, indicating that the CSSLs were suitable for genetic mapping research. The correlation between seed-pod-related traits was analyzed. PoOSPN and PoTSPN were positively correlated; PoTHSPN and PoFSPN were positively correlated; PoOSPN were also negatively correlated with PoTHSPN and PoFSPN; and PoTSPN were also negatively correlated with PoTHSPN and PoFSPN. SL was positively correlated with SW, SL and SW were positively correlated with HSW. SL, SW, and HSW were significantly positively correlated with SPW. The above results showed that the pod-seed-related traits of soybean were not isolated, and the traits were closely related (Supplementary Table 3).

### Screening Materials for Further Construction of CSSLs

**Figure 3:**
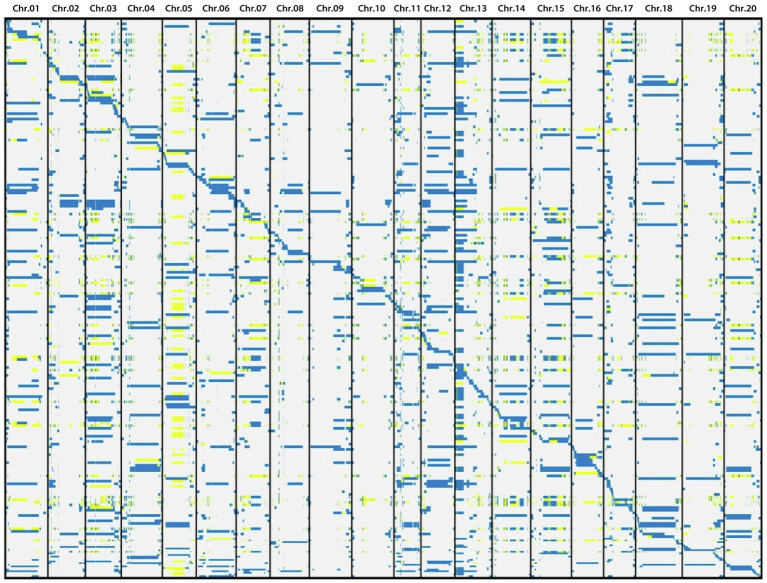
Schematic diagram of introgressed fragments of 129 lines. Blue, white and yellow represent ZYD00006, suinong14 and heterozygote genotypes, respectively.

**Figure 4:**
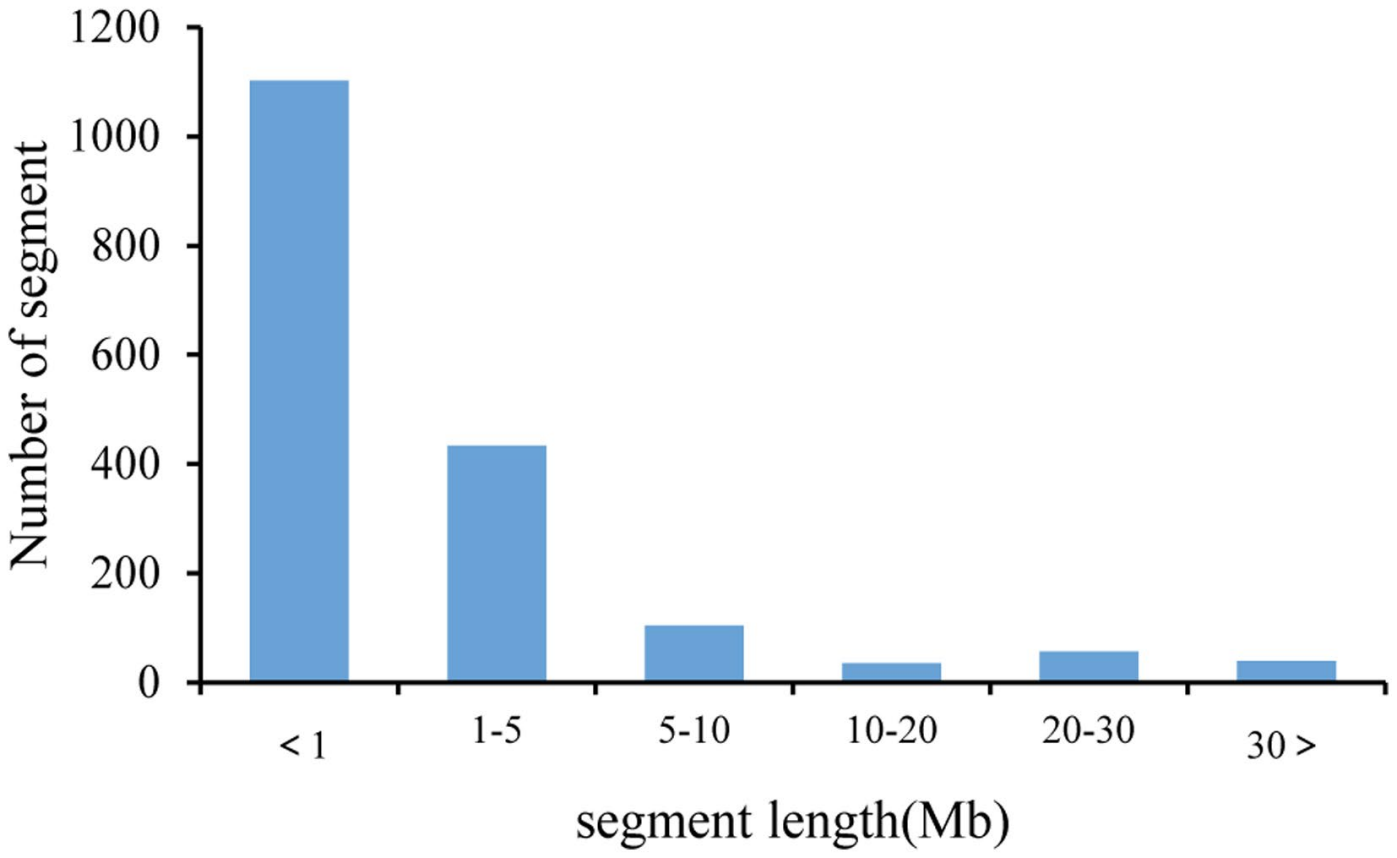
Frequency of substituted chromosome segments according to length.

Based on the information of sequencing and introduction of donor fragments, 129 lines which can be achieve the maximum coverage of CSSLs were selected from 213 CSSLs as the basis for future construction of CSSLs (Figure 3). These 129 CSSL carried 1772 introgressed segments (including 359 introgressed segments with a single bin marker; Figure 4). The average number of introgressed segments per chromosome in these lines was 88.6. The length of these introgressed segments ranged from 0.02 Mb to 47.14 Mb, and their average length was 3.3 Mb. Among the above introgressed segments, 62.19% of the introgressed segments were less than 1 Mb, 24.49% were between 1 and 5 Mb, and large segments greater than 10 Mb accounted for only 7.45%. The total length of introgressed segments in the CSSLs was 5589.34 Mb, which was 5.71 times larger than the soybean genome and covered 89.65% of the genome of wild soybean (Table 4). In the future, we will further expand the CSSLs on this basis of 129 lines to complete the construction of whole-genome substitution lines.

**Table 4:** The number and coverage of introgressed fragments per chromosome in CSSLs.

Chromosome	Introgressed segments	Maximum (Mb)	Minimum (Mb)	Average segment length (Mb)	Coverage (%)
1	64	42.10	0.02	5.34	84.90
2	116	22.33	0.02	2.30	86.58
3	126	31.91	0.03	3.53	87.27
4	90	30.93	0.02	3.03	89.14
5	64	33.84	0.02	4.22	90.31
6	83	39.71	0.03	3.54	88.01
7	88	33.76	0.02	2.55	84.83
8	73	29.34	0.02	2.67	92.72
9	52	35.76	0.03	2.91	96.54
10	66	35.36	0.03	3.03	92.41
11	112	22.37	0.02	1.97	85.60
12	64	35.10	0.02	5.22	94.72
13	135	24.98	0.02	1.63	90.89
14	72	37.53	0.02	3.45	88.71
15	117	44.23	0.02	3.78	88.61
16	57	25.81	0.02	3.49	95.08
17	103	16.50	0.04	2.37	87.16
18	85	47.14	0.03	5.63	91.14
19	113	40.24	0.02	2.06	92.61
20	92	31.87	0.02	3.37	86.17
Average	88.60	33.04	0.02	3.30	89.65

### The QTL Mapping of Seed-Pod Traits

**Figure 5:**
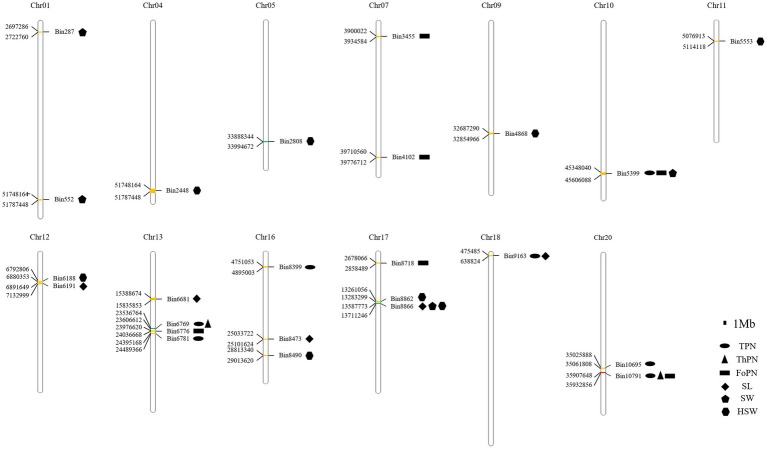
The QTLs were detected steadily for more than 2 years. The QTLs detected steadily for 2 years were yellow; The QTLs detected steadily for 2 years were green; The QTLs detected steadily for 5 years were red.

Based on the bin data from CSSLs and the phenotypic data from 8 seed-pod-related traits of CSSLs for 5 consecutive years and BLUE of 5 years, 170 QTLs were detected by QTL analysis (Table 5; Supplementary Figure 1; Supplementary Table 4). Thirty-two QTLs were detected steadily for more than 2 years. Among them, 7, 2, 6, 5, 4, and 8 QTLs were related to the PoTSPN, PoTHSPN, PoFSPN, SL, SW and HSW, respectively (Figure 5, Table 5). The PoOSPN and SPW were no QTL detected steadily for more than 2 years. Compared with total QTLs mapped for each trait, 23, 7, 1, and 1 QTLs were repeatedly detected in 2, 3, 4, and 5 traits, respectively.

**Table 5:** The QTLs were detected steadily in CSSLs for more than 2 years.

QTL	Year	Chromosome	Marker name	LOD	PVE (%)	Add
*qPoTSPN13-2*	2016	Chr13	Bin6781	3.58	5.82	−0.04
*qPoTSPN13-2*	2019	Chr13	Bin6781	3.8	5.05	−0.03
*qPoTSPN13-1*	2017	Chr13	Bin6769	9.17	13.6	−0.06
*qPoTSPN13-1*	2018	Chr13	Bin6769	12.48	15.71	−0.06
*qPoTSPN13-1*	2020	Chr13	Bin6769	10.7	13.89	−0.07
*qPoTSPN13-1*	BLUE	Chr13	Bin6769	18.77	20.48	−0.06
*qPoTSPN16-1*	2018	Chr16	Bin8399	4.48	5.19	0.05
*qPoTSPN16-1*	2019	Chr16	Bin8399	11.92	17.51	0.07
*qPoTSPN18-1*	2016	Chr18	Bin9163	2.57	3.89	0.16
*qPoTSPN18-1*	2017	Chr18	Bin9163	3.27	4.15	0.16
*qPoTSPN20-1*	2017	Chr20	Bin10695	3.04	4.3	0.07
*qPoTSPN20-1*	2019	Chr20	Bin10695	8.26	11.8	0.11
*qPoTSPN20-3*	2018	Chr20	Bin10791	12.7	15.83	−0.08
*qPoTSPN20-3*	2020	Chr20	Bin10791	7.98	9.89	−0.07
*qPoTSPN20-3*	BLUE	Chr20	Bin10791	16.34	17.29	−0.06
*qPoTHSPN13-1*	2017	Chr13	Bin6769	4.18	6.26	0.05
*qPoTHSPN13-1*	2018	Chr13	Bin6769	7.64	11.95	0.04
*qPoTHSPN13-1*	BLUE	Chr13	Bin6769	16.67	16.14	0.05
*qPoTHSPN20-1*	2017	Chr20	Bin10791	6.26	9.46	−0.07
*qPoTHSPN20-1*	BLUE	Chr20	Bin10791	16.32	15.42	−0.05
*qPoFSPN7-1*	2017	Chr07	Bin3455	3.46	3.87	0.02
*qPoFSPN7-1*	BLUE	Chr07	Bin3455	2.96	1.94	0.02
*qPoFSPN7-2*	2017	Chr07	Bin4102	7.19	8.76	0.02
*qPoFSPN7-2*	BLUE	Chr07	Bin4102	10.5	7.44	0.02
*qPoFSPN10-1*	2019	Chr10	Bin5399	6.35	9	−0.03
*qPoFSPN10-1*	BLUE	Chr10	Bin5399	8.39	5.74	−0.02
*qPoFSPN13-1*	2018	Chr13	Bin6776	4.22	6.69	0.02
*qPoFSPN13-1*	BLUE	Chr13	Bin6776	12.63	9.18	0.02
*qPoFSPN17-1*	2018	Chr17	Bin8718	3.32	5.14	−0.03
*qPoFSPN17-1*	BLUE	Chr17	Bin8718	4.65	3.05	−0.02
*qPoFSPN20-3*	2017	Chr20	Bin10791	6.94	8.35	−0.02
*qPoFSPN20-3*	2018	Chr20	Bin10791	9.13	15.12	−0.04
*qPoFSPN20-3*	2019	Chr20	Bin10791	12.32	18.8	−0.04
*qPoFSPN20-3*	2020	Chr20	Bin10791	4.36	7.69	−0.04
*qPoFSPN20-3*	BLUE	Chr20	Bin10791	33.31	30.69	−0.05
*qSL12-2*	2018	Chr12	Bin6191	95.75	20.09	3.39
*qSL12-2*	BLUE	Chr12	Bin6191	3.41	4.73	0.13
*qSL13-1*	2016	Chr13	Bin6681	4.83	10.38	−0.26
*qSL13-1*	2017	Chr13	Bin6681	5.17	7.5	−0.27
*qSL16-1*	2017	Chr16	Bin8473	4.96	6.91	−0.26
*qSL16-1*	BLUE	Chr16	Bin8473	6.34	8.77	−0.18
*qSL17-1*	2017	Chr17	Bin8866	10.91	16.93	0.36
*qSL17-1*	BLUE	Chr17	Bin8866	17.58	28.76	0.30
*qSL18-1*	2017	Chr18	Bin9163	6.17	8.99	1.04
*qSL18-1*	2020	Chr18	Bin9163	9.1	14.25	0.94
*qSW1-2*	2018	Chr01	Bin287	5.3	8.04	−0.15
*qSW1-2*	BLUE	Chr01	Bin287	6.08	9.9	−0.11
*qSW1-6*	2020	Chr01	Bin522	6.08	10.54	0.30
*qSW1-6*	BLUE	Chr01	Bin522	4.99	8.09	0.23
*qSW10-2*	2018	Chr10	Bin5399	6.52	10.01	−0.24
*qSW10-2*	BLUE	Chr10	Bin5399	8.42	14.09	−0.19
*qSW17-1*	2018	Chr17	Bin8866	10.3	16.54	0.30
*qSW17-1*	2019	Chr17	Bin8866	5.78	9.92	0.24
*qSW17-1*	BLUE	Chr17	Bin8866	6.27	10.25	0.16
*qHSW4-1*	2020	Chr04	Bin2448	3.04	2.24	−0.60
*qHSW4-1*	BLUE	Chr04	Bin2448	3.05	2.26	−0.60
*qHSW5-2*	2017	Chr05	Bin2808	36.73	22.8	−3.28
*qHSW5-2*	2018	Chr05	Bin2808	3.02	3.25	−0.93
*qHSW5-2*	2019	Chr05	Bin2808	5.09	4.84	−1.15
*qHSW9-2*	2017	Chr09	Bin4868	10.18	4.38	−1.35
*qHSW9-2*	BLUE	Chr09	Bin4868	6.68	4.78	−1.02
*qHSW11-2*	2020	Chr11	Bin5553	5.02	4.11	−0.67
*qHSW11-2*	BLUE	Chr11	Bin5553	10.3	8.16	−0.97
*qHSW12-3*	2020	Chr12	Bin6188	5.92	4.76	−1.08
*qHSW12-3*	BLUE	Chr12	Bin6188	6.01	2.52	−0.89
*qHSW16-2*	2020	Chr16	Bin8490	3.41	2.73	−1.08
*qHSW16-2*	BLUE	Chr16	Bin8490	3.43	2.75	−1.09
*qHSW17-3*	2020	Chr17	Bin8862	33.96	37.46	3.30
*qHSW17-3*	BLUE	Chr17	Bin8862	34.47	33.13	3.12
*qHSW17-4*	2017	Chr17	Bin8866	26.62	14.08	2.70
*qHSW17-4*	2018	Chr17	Bin8866	16.24	21.07	2.41
*qHSW17-4*	2019	Chr17	Bin8866	14.86	16.23	2.21

Among the QTLs that were detected steadily for more than 2 years, QTLs with the highest proportions of phenotypic variation explained were *qPoTSPN13-1*, *qPoTHSPN13-1*, *qPoFSPN20-3*, *qSL17-1*, *qSW17-1*, and *qHSW17-3*, respectively (Table 5). *qPoTSPN13-1* and *qPoTHSPN13-1* are the same locus detected on chromosome 13, and the proportions of phenotypic variation explained were 20.48 and 16.14%, respectively. *qPoFSPN20-3* is a QTL detected on chromosome 20, and the proportion of phenotypic variation explained was 30.69%. *qSL17-1* and *qSW17-1* are the same locus detected on chromosome 17, and the proportions of phenotypic variation explained were 28.76 and 16.54%, respectively. *qHSW17-3* is a QTL detected on chromosome 17, and the proportion of phenotypic variation explained was 37.58%. When comparing QTLs related to different traits that were stably detected for more than 2 years, we found that two QTLs, *qPoTSPN18-1* and *qSL18-1*, were detected at locus Bin9163; three QTLs, *qPoTSPN10-1, qPoFSPN10-1*, and *qSW10-2*, were detected at locus Bin5399; two QTLs, *qPoTSPN13-1* and *qPoTHSPN13-1*, were detected at locus Bin6769; three QTLs, *qPoTSPN20-3, qPoTHSPN20-1*, and *qPoFSPN20-3*, were detected at locus Bin10791; and the locus Bin8866 was detected as *qSL17-1, qSW17-1* and *qHSW17-4* (Figure 5). Compared with the QTL detected only once, the QTL repeatedly mapped between different traits or between different years of the same trait has higher feasibility. The above QTLs will be the focus of further research.

### Establishment of a Secondary Segregating Population and Fine Mapping

**Figure 6:**
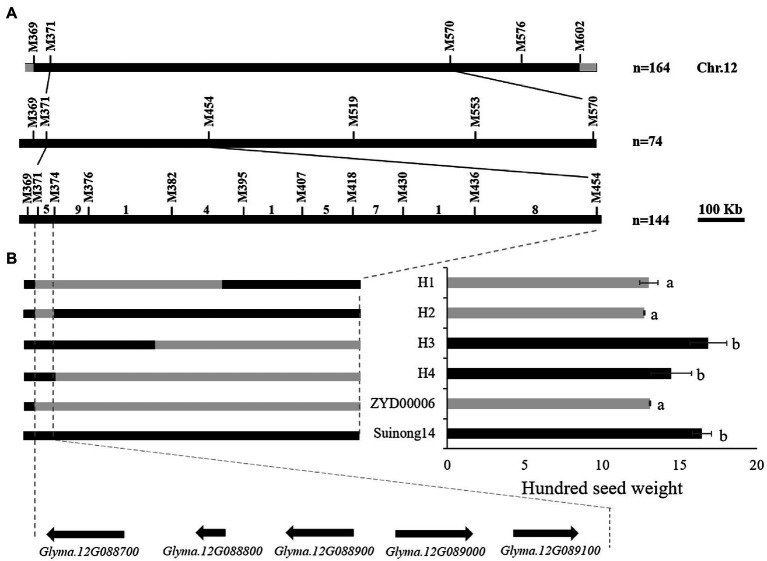
QTL fine mapping. **(A)** The *qHSW-12-3* locus was detected on chromosome 12. Positional mapping narrowed the *qHSW-12-3* locus to a 64.4 kb region between M371 and M374. The numbers above the bars indicated the number of recombinants. **(B)** Fine-map the genotype of the population and the significance of the corresponding phenotype. Gray indicates Suinong14 genotype, black indicates ZYD00006 genotype. a, b: Different letters represent significant differences between each other at the 0.01 level.

R183 was a material in CSSLs with extremely small HSW; several HSW QTLs stably detected more than 2 years were located in the introgressed segment of R183 (bin2808, bin5553, and bin6188). The secondary segregating population was constructed by backcrossing R183 (12.74 g) with SN14 (19.73 g; Supplementary Figures 2A,B) to identify candidate genes regulating soybean HSW. Forty-five pairs of SSR markers with polymorphisms between parents were screened. Preliminary mapping of QTLs employing the secondary segregating population R183-F_2_ and the 45 SSR markers detected 5 QTLs (Supplementary Figure 2C; Table 6). Among them, *qHSW-12-1* was located between two flanking markers, M371 (7.27 Mb) and M570 (11.68 Mb). *qHSW-12-1* had the highest proportion of phenotypic variation explained (21.42%). Next, fine mapping of *qHSW-12-1* was performed (Figure 6A).

**Table 6:** QTLs Preliminary mapping of the secondary segregating population.

Chromosome	Left-marker	Right-marker	LOD	PVE (%)	Add
Chr02	M257	M319	5.75	10.75	−0.55
Chr03	M491	M520	4.82	11.94	0.42
Chr11	M135	M274	5.40	10.04	−0.49
Chr12	M371	M570	10.86	21.42	−0.73
Chr17	M330	M350	4.20	7.87	−0.42

One heterozygous plant in the target interval was screened from the secondary segregating population R183-F_2_, and 74 R183-F_2:3_ plants were obtained to narrow the target interval. Fine mapping of *qHSW-12-1* was performed by the secondary segregating population R183-F_2:3_ and 5 SSR markers in the target interval. *qHSW-12-1* was mapped between two flanking markers, M371 (7.27 Mb) and M454 (8.50 Mb; Supplementary Figures 2D,E). The fine mapping QTL interval was larger, so individuals with heterozygous target segments were selected to construct the RHL population, and a total of 144 individuals were obtained. Ten SSR markers were employed to increase the density of primers in the target interval. Finally, *qHSW-12-1* was narrowed down to a 64 kb region between M371 (7.27 Mb) and M374 (7.33 Mb; Figure 6B; Supplementary Figures 2F,G).

**Figure 7:**
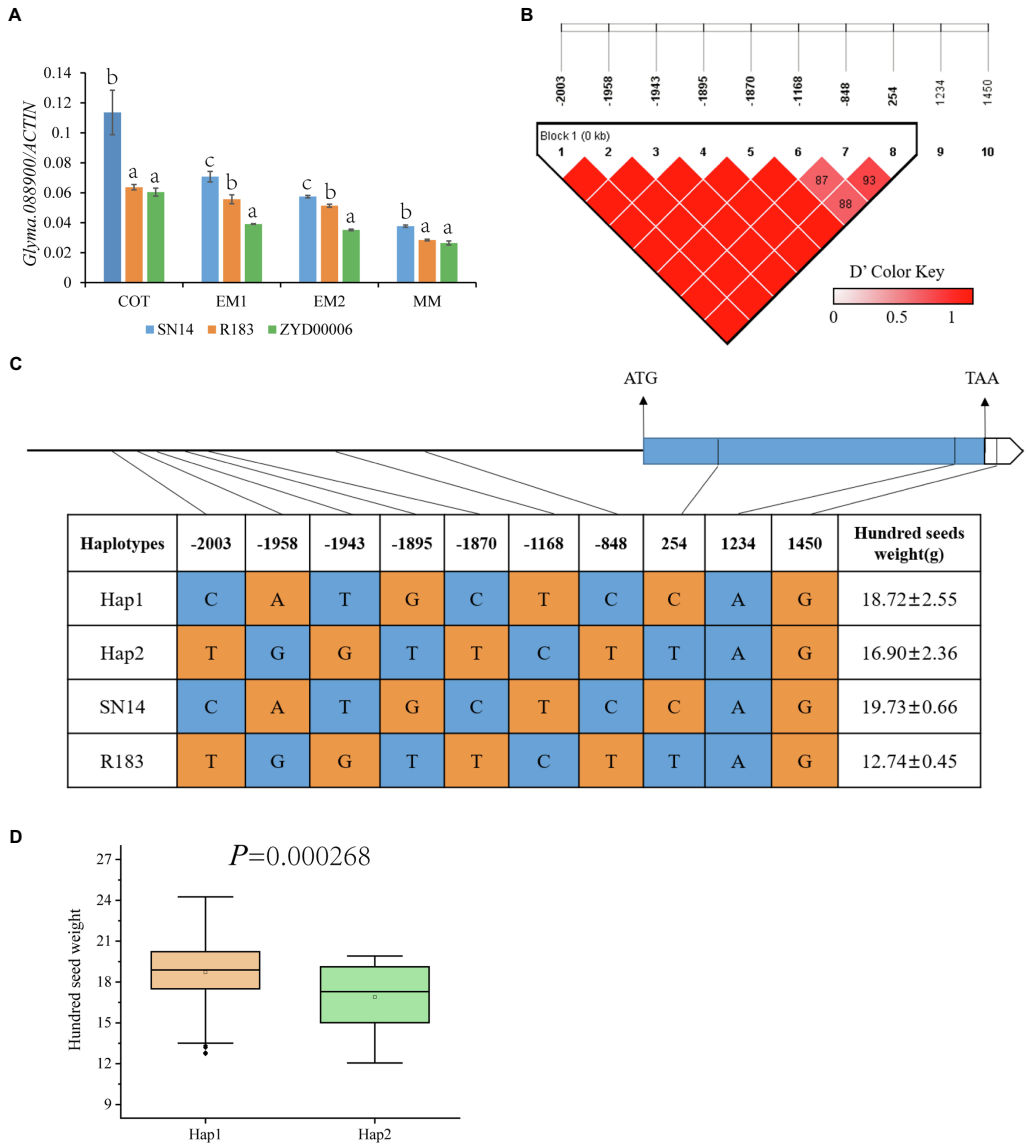
Inferred candidate gene. **(A)** The relative expression of *Glyma.12G088900* in SN14, ZYD00006 and R183 was determined by qRT-PCR. **(B)** LD analysis of SNPs located on *Glyma.12G088900*. **(C)** Haplotype analysis of *Glyma.12G088900* from 129 soybean resource. **(D)** The hundred-seed weight (HSW) of Hap1 and Hap2. a, b, c: Different letters represent significant differences between each other at the 0.05 level.

According to the Williams 82 soybean reference genome sequence in the Phytozome database (See Footnote 4), the genome interval of *qHSW-12-1* contains a total of 5 genes (*Glyma.12G088700*, *Glyma.12G088800*, *Glyma.12G088900*, *Glyma.12G089000*, and *Glyma.12G089100*; Figure 6B). *Glyma.12G088700*, a transcription factor related to drought resistance and salt tolerance (Liao et al., 2008; Li et al., 2019), showed no difference between the parents in the sequences of the upstream 2000 bp promoter or the CDS region. *Glyma.12G088800* has no genetic annotation, and quantitative real-time PCR (qRT-PCR) showed that the gene was not expressed during seed development periods. *Glyma.12G089000* (annotated as transmembrane 9 superfamily member 5) is a gene that has not been studied at present. qRT-PCR showed that there was a significant difference in gene expression between parents at the COT and EM1 stages, but there was no significant difference at the EM2 and MM stages. *Glyma.12G089100* is also called *GmPHR20*. Its homologous gene (*GmPHR25*) in soybean is an important regulator of the phosphorus signal network (Xue et al., 2017), and its homologous gene (*AT3G04030*) in Arabidopsis may be involved in nitrogen reuse (Nakano et al., 2017). However, qRT-PCR showed that there was no difference in the relative expression of the gene between parents. There were 7 SNP differences in the promoter region and 2 SNP differences in the CDS region in *Glyma.12G088900* between parents (Supplementary Figures 3–5). qRT-PCR showed that there were extremely significant differences in the gene during the seed development periods of both parents (Figure 7A; Supplementary Table 5). Dnasp5.0 software was used to analyze the haplotype distribution of *Glyma.12G088900* SNP sequences in germplasm resource populations. There was an extremely significant difference in HSW between two excellent haplotypes, Hap1, with the same genotype as R183, and Hap2, with the same genotype as SN14, indicating that *Glyma.12G088900* may be a gene regulating HSW (Figures 7B–D; Supplementary Table 6).

### Analysis of Epistasis Between Loci

**Figure 8:**
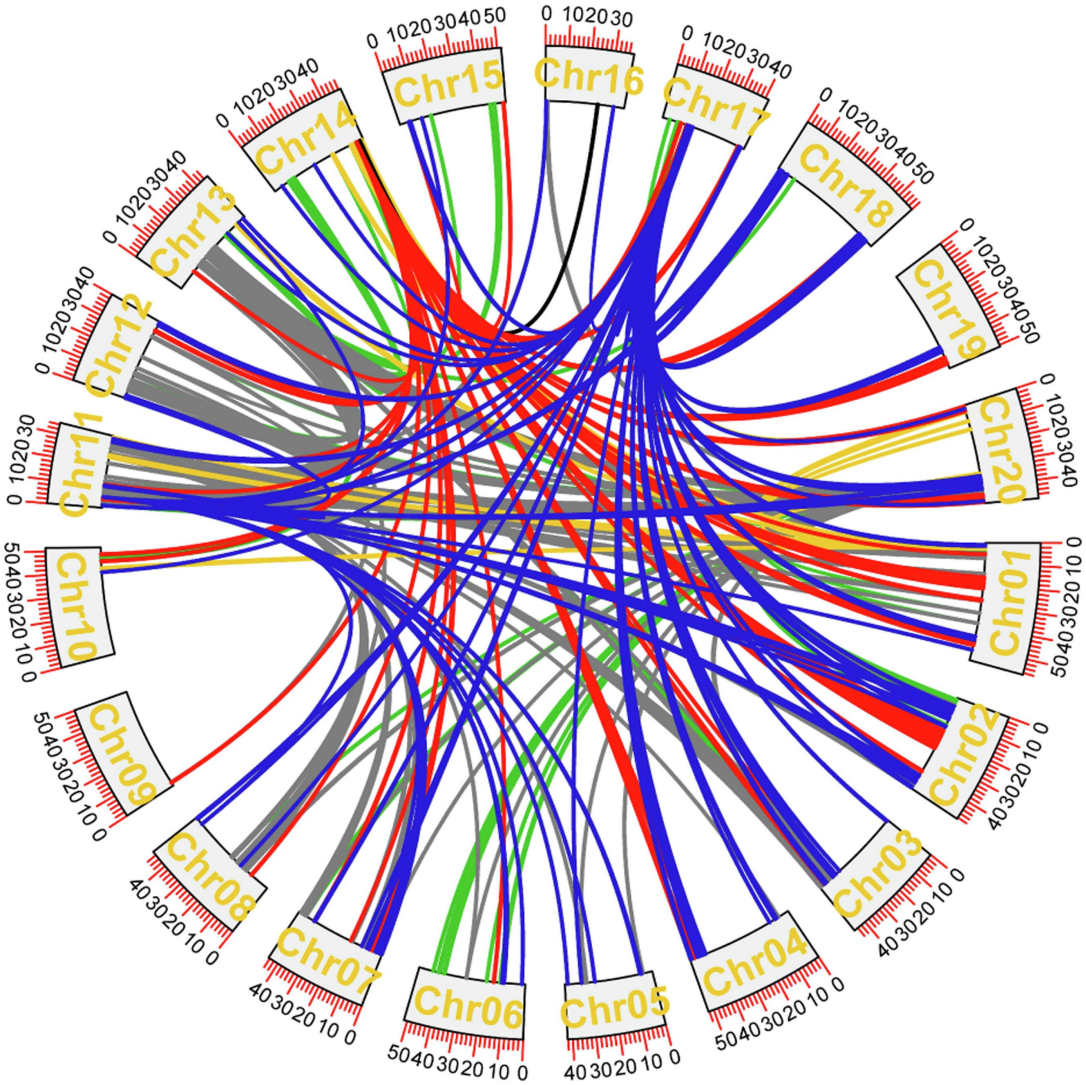
Epistasis analysis of CSSLs. Epistasis of seed-pod traits that have repeatedly appeared over the years. Dark blue represents the epistatic effect among HSW loci, red represents the epistatic effect among PoOSPN loci, black represents the epistatic effect among SL loci, yellow represents the epistatic effect among SW loci, green represents the epistatic effect among PoTHSPN loci, gray represents the epistatic effect among PoTSPN loci.

The epistatic effects were analyzed by the BLUE values of 8 traits in 5 years. The results showed that 955 pairs of epistatic QTLs were obtained from the other 6 traits except PoFSPN and SPW (Figure 8; Supplementary Table 7). Among these epistatic QTLs, the LOD values were between 10.01 and 36.08, and the proportions of phenotypic variation explained were between 0.11 and 12.85%. There were 171, 79, 6, 38, 282, and 379 pairs of epistasis in HSW, PoOSPN, SL, SW, PoTSPN, and PoTHSPN, respectively. Among them, the maximum proportions of phenotypic variation explained were 0.63, 0.46, 12.85, 1.60, 0.53, and 0.35%, respectively, and the minimum proportions were 0.14, 0.28, 5.52, 1.12, 0.16, and 0.11%, respectively. Except for SL, the proportions of phenotypic variation explained of epistasis about other traits were lower, indicating that the main effect QTL played an important role in the regulation of target traits.

In HSW, 19 pairs of epistasis occurred between two major QTL, the other occurred between a major QTL and a locus, and a total of 129 pairs played a positive role in increasing HSW. *qHSW17-3* not only had epistatic interaction with 5 major QTL (*qHSW3-2, qHSW5-3, qHSW4-1, qHSW18-2, qHSW20-3*), but also had epistatic interaction with 110 motifs, indicating that the QTL may play an important role in the regulation of HSW.

Seventy-eight pairs of epistasis related to PoOSPN occurred between one major QTL and one locus, only one pair of epistasis occurred between two major QTL. Among them, 59 pairs played a positive role in reducing PoOSPN. All the above 79 pairs of epistasis were related to *qPoOSPN14-1*, indicating that qPoOSPN14-1 played an important role in regulating PoOSPN.

Six pairs of epistasis related to SL occurred between one major QTL and one locus, in which the pairs of epistasis between *qSL16-1* and 2 loci had negative effects on SL increase and the pairs of epistasis between *qSL17-1* and 4 loci played positive roles in SL increase. The results showed that *qSL17-1* may play an important role in regulating SL.

Thirty-eight pairs of epistatic effects related to SW occurred between a major QTL and a locus, in which 20 pairs of epistasis had negative effects on the increase of SW. There were epistatic interactions between *qSW1-2* and 34 loci, indicating that *qSL17-1* may play an important role in regulating SW.

Eighty-eight pairs of epistasis related to PoTSPN occurred between two major QTL, and the other occurred between one major QTL and one locus. A total of 261 pairs of epistatic played positive roles in reducing PoTSPN. *qPoTSPN11-1, qPoTSPN13-1* and *qPoTSPN20-3* not only had epistatic interactions with 6, 4, and 4 major QTLs, respectively, but also had epistatic interactions with 14, 27, and 28 loci, respectively, indicating that the three QTLs may play important roles in regulating PoTSPN.

One hundred sixty pairs of epistasis related to PoTHSPN occurred between two major QTLs, and the other occurred between one major QTL and one locus. *qPoTHSPN11-1, qPoTHSPN13-1*, and qPoTHSPN20-1 had epistatic interactions not only with 4, 2, and 4 major QTLs, respectively, but also with 51, 56, and 45 loci, respectively, indicating that the three QTLs may play important roles in regulating PoTHSPN.

## Discussion

Wild soybean was domesticated approximately 6,000–9,000 years ago (Zhou et al., 2015), and is recognized as the wild ancestor of cultivated soybean (Zhang et al., 2018). In the process of domestication, due to purposeful selection by human beings, the genetic diversity of cultivated soybean became much lower than that of wild soybean (Hyten et al., 2006; Lam et al., 2010). In this study, CSSLs were constructed between cultivated soybean SN14 and wild soybean ZYD00006. Through multigeneration backcrossing, the introduction of the wild soybean genome greatly enriched the genetic base of cultivated soybean, which led to rich phenotypic variation in the CSSLs. For example, the maximum HSW is 25.69 g, the minimum is 9.83 g; the maximum SPW is 38.68 g, the minimum is 11.62 g; and the maximum PoTSPN is 59.06%, the minimum is 15.08% (Figure 2; Supplementary Table 1). In this study, we also employed SNP markers to divide the soybean genome into 3,780 bin markers. High-density bin markers can improve the efficiency and accuracy of QTL mapping, which makes it possible to perform fine mapping of QTLs and gene mining. In the next step of research, 129 lines covering 89.65% of wild soybean genome were selected from CSSLs and hybridized with SN14. MAS was used to shorten the introgressed fragments, purify the genetic background of the CSSLs and improve the genome coverage of wild soybean. Finally, we will complete the construction of whole-genome substitution lines so that they have greater utilization value.

In order to increase the accuracy of QTL mapping, we integrated the phenotypes in different years to calculate the BLUE value of each trait. BLUE, derived from the linear mixed model, has been popularly used to estimate animal and plant breeding values for a few decades (Kim, 2020). BLUE can more effectively eliminate the deviation caused by the environment and improve the accuracy of QTL mapping. Muqaddasi et al. (2017) studied anther extrusion of wheat accessions by genome-wide association studies with BLUE of anther extrusion. Then, they predicted the candidate genes which could be directly or indirectly involved in anther extrusion. Li et al. (2019) employed the BLUE of soybean plant height and the number of nodes on the main stem for QTL mapping, and predicted two candidate genes for simultaneous regulation of these two traits. In addition, we selected appropriate method to improve the accuracy of QTL mapping. Because most CSSLs used for QTL mapping are non-idealized CSSLs (There is only one import fragment in each CSSL.; Li et al., 2015; Bux et al., 2019). There are redundant markers, and there may be multicollinearity between markers. The goal of QTL mapping is to explore the impact of different markers on target traits and quantitatively describe their impact on target traits. Multicollinearity between markers will reduce the positioning ability and calculation accuracy, so multicollinearity must be controlled. Wang et al. (2006) developed RSTEP-LRT model for QTL mapping of non-idealized CSSLs. Firstly, the model will evaluate the multicollinearity between different markers and delete redundant markers. However, there is no theoretical method to find an appropriate threshold to calculate whether the multicollinearity is high. In this study, we used the condition number −1,000 as the threshold of multicollinearity, which was one preset value in the software. It is equivalent to deleting duplicate markers, to reduce the adverse effect of multicollinearity on QTL mapping.

QTL mapping is based on the difference in natural alleles among parent varieties (Ma et al., 2019), and its mapping accuracy is limited by the mapping population. CSSLs can eliminate background interference during QTL mapping and maintain high mapping accuracy when QTLs are located on very small fragments. In recent years, CSSLs have been widely used in QTL mapping and candidate gene mining (Balakrishnan et al., 2019). Huo et al. (2017) used CSSLs to fine map the number of grains per panicle and verify the function of the candidate gene *NOG1*. Yang et al. (2017) fine mapped soybean HSW, protein content, and oil content using CSSLs. The bidirectional sucrose transporter gene *Glyma.15 g049200* was predicted to be a pleiotropic regulatory gene that regulates HSW, protein content, and oil content. In our study, QTL mapping of seed-pod traits in wild soybean was carried out by CSSLs of wild soybean. Eight QTLs related to HSW were identified for more than 2 years, of which four QTLs coincided with previous mapping results (Hyten et al., 2004; Gai et al., 2007; Kuroda et al., 2013), namely *qHSW9-2, qHSW11-2, qHSW17-3*, and *qHSW17-3*. Five-seed length QTLs and four seed width QTLs were reported steadily for more than 2 years, and two of them were seed width QTLs located on chromosome 1 for the first time. *qPoTSPN20-3*, *qPoTHSPN20-1*, and *qPoFSPN20-3* are the same locus detected on chromosome 20. This is the only QTL detected that has been stable for more than 2 years and is related to the POTSPN, PoTHSPN, and PoFSPN. Compared with the results of previous studies, we found that the QTL detected on chromosome 20 coincided with the cloned *Ln* (Sayama et al., 2017), and *Ln* had been demonstrated to have a polymorphism to control the leaf shape and pod number for soybean. The above results reflect the accuracy and effectiveness of this set of CSSL materials in determining the positions of QTLs.

To explore the excellent alleles regulating HSW, we constructed a secondary segregation population by backcrossing CSSL-R183 (which had an extremely small HSW) with SN14. The HSW QTL was mapped to the 64.4 Kb region of chromosome 12 by two fine mapping. *Glyma.12G088900* was preliminarily identified as a candidate gene by sequence analysis of genes in the 64.4 kb region, relative expressivity analysis at important developmental stages, and haplotype analysis. *Glyma.12G088900* belongs to the F-box/LRR family of proteins, which can regulate a variety of activities, such as regulating plant floral organ development, responding to biotic and abiotic stresses, and regulating growth, development and crop yield (Chen et al., 2013; Jia et al., 2015; Baute et al., 2017; He et al., 2017). At the same time, the homologous gene in Arabidopsis (*AT1G67190*) can interact with SKP1-like protein to form the SCF complex and regulate grain development, in which *ASK1* and *ASK2* have the function of regulating embryogenesis and seed development (Liu et al., 2004; Kuroda et al., 2012).

Many studies have shown that epistatic effects were important factors affecting agronomic traits of soybean and important genetic components of quantitative trait variation, and the epistatic effects between major QTLs had very important effects on quantitative traits (Jiang et al., 2018). Qi et al. (2017) used RIL and CSSLs to carry out QTL mapping and epistatic analysis of soybean oil traits, and a total of 11 QTLs and 4 pairs of epistatic QTLs were detected, which provided important information for soybean molecular-assisted breeding. Balakrishnan et al. (2019) used CSSLs to carry out QTL mapping and epistatic analysis. A total of 22 QTLs and 17 pairs of significant epistatic QTLs for 11 traits were detected, which provided valuable resources for gene discovery and yield improvement. In this study, 955 pairs of epistatic QTLs were detected by CSSLs to analyze seed-pod traits of soybean. Among them, *qHSW17-3, qPoOSPN14-1, qPoTSPN11-1, qPoTSPN13-1, qPoTSPN20-3, qPoTHSPN11-1*, and *qPoTHSPN20-1* have epistatic interactions not only with other major QTLs, but also with a large number of loci. But the proportions of phenotypic variation explained of above epistasis were low, the highest is only 0.62%. However, when a large number of epistatic effects were aggregated, it was still an important part that cannot be ignored in actual production. For breeding programs to improve seed-pod traits, in addition to the most important process of aggregating reliable QTLs (especially the main QTLs), attention should also be paid to the loci and interaction effects that interact with main QTLs, then the QTLs can make the greatest contribution to target traits (Jiang et al., 2011). In addition, bin5858 (*qPoTSPN11-1, qPoTHSPN11-1*) had epistasis interactions with bin6769 (*qPoTSPN13-1, qPoTHSPN13-1*) and *bin10791 (qPoTSPN20-3, qPoTHSPN20-1*) in PoTSPN, PoTHSPN, respectively. Interestingly, the two pairs epistasis resulted in a decrease in PoTSPN, while an increase in PoTHSPN. The results showed that seed-pod traits were often closely related, and the same locus can not only directly affect different pod characters, but also indirectly regulate them through epistasis (Fang et al., 2017; Yang et al., 2021). The loci controlling several pod traits and the loci with a large number of epistasis found in this study can provide valuable information for molecular-assisted breeding of soybean.

## Conclusion

In this study, 580,542 SNPs were identified between SN14 and ZYD00006, and were divided into 3,780 bin markers. A total of 3,780 bin markers were used for QTL mapping of seed-pod-related traits in CSSLs. A total of 170 QTLs were detected, of which 32 QTLs appeared stably in different years. A secondary segregation population was constructed for HSW, and *qHSW-12-1* was mapped to the 64.4 Kb region of chromosome 12. qRT-PCR showed that there were significant differences in the gene during seed development in the two parents. Further analysis showed that there was a difference in *Glyma.12G088900* in the promoter region and an amino acid difference in the coding region, which may affect HSW. A total of 955 pairs of epistatic sites were detected in epistatic analysis. Among them, 161 QTL mapped by CSSLs have epistatic effect. The construction of wild soybean CSSLs provides excellent genetic materials for broadening the genetic base of cultivated soybean and mining important candidate genes, and the genetic study of related traits lays a theoretical foundation for the study of seed-pod traits in soybean.

## Data Availability Statement

The datasets presented in this study can be found in online repositories. The names of the repository/repositories and accession number(s) can be found in the article/Supplementary Material.

## Author Contributions

CL, HJ, and QC conceived the study and designed and managed the experiments. MY, CL, and ZQ provided plant lines. HZ, FC, and RW performed trials and collected data. ZZ, RZ, DX, and HZ completed statistical analyses of phenotypic data. HZ, LH, and JX contributed to writing the paper. All authors contributed to the article and approved the submitted version.

## Funding

This study was financially supported by the National Nature Science Foundation of China (U20A2027 and 31771882), China Agriculture Research System of MOF and MARA (CARS-04-PS07 and CARS-04-PS11), National Key R&D Program of China (2021YFF1001202), Hundred-thousand and million project of Heilongjiang province for engineering and technology science’ soybean breeding technology innovation and new cultivar breeding (2019ZX16B01-1), Funding Scheme for Introducing High-level Scientific and Technological Innovation Talents by Jilin Research Institutes in 2019.

## Conflict of Interest

The authors declare that the research was conducted in the absence of any commercial or financial relationships that could be construed as a potential conflict of interest.

## Publisher’s Note

All claims expressed in this article are solely those of the authors and do not necessarily represent those of their affiliated organizations, or those of the publisher, the editors and the reviewers. Any product that may be evaluated in this article, or claim that may be made by its manufacturer, is not guaranteed or endorsed by the publisher.

## Supplementary Material

The Supplementary Material for this article can be found online at: https://www.frontiersin.org/articles/10.3389/fpls.2022.869455/full#supplementary-material

Supplementary Figure 1 QTLs that mapped only in a single year.

Supplementary Figure 2 The fine mapping of *qHSW12-3*. **(A)** Phenotypic of ZYD00006 and the parents of the secondary segregation. **(B)** The hundred-seed weight (HSW) of ZYD00006 and the parents of the secondary segregation. **(C)** The QTL mapping result of R183-F_2_. **(D)** The frequency chart of F_2:3_. **(E)** The QTL mapping result of R183-F_2:3_. **(F)** The frequency chart of RHL. **(G)** The QTL mapping result of RHL.

Supplementary Figure 3 Sequence comparison of promoter.

Supplementary Figure 4 Sequence comparison of transcript sequence.

Supplementary Figure 5 Sequence comparison of Protein sequence.

## Footnotes

^1^ https://soybase.org/

^2^ https://soybase.org/amigo/amigo/term/SOY:0001283#display-lineage-tab

^3^ http://www.premierbiosoft.com/primerdesign/index.html

^4^ https://phytozome.jgi.doe.gov/pz/portal.html

^5^ https://haploview.software.informer.com/4.2/

^6^ https://www.dna.affrc.go.jp/PLACE/

^7^ http://bioinformatics.psb.ugent.be/webtools/plantcare/html/

## Abbreviations

CSSLs: Chromosome Segment Substitution Lines
MAS: Marker-Assisted Selection
SNP: Single-Nucleotide Polymorphisms
ICIM: Inclusive Composite Interval Mapping
PoOSPN: The Proportion of One-Seed-Pod-Numbers
PoTSPN: The Proportion of Two-Seed-Pod-Numbers
PoTHSPN: The Proportion of Three-Seed-Pod-Numbers
PoFSPN: The Proportion of Four-Seed-Pod-Numbers
SL: Seed Length
SW: Seed Width
HSW: Hundred-Seed Weight
SPW: Seed-Pod Weight
QTL: Quantitative Trait Loci
LOD: Logarithm of Odds
PVE: Phenotypic Variance Explained
qRT-PCR: Quantitative Real-time PCR
