# Supplementary figures and images for: Construction of Chromosome Segment Substitution Lines and Inheritance of Seed-Pod Characteristics in Wild Soybean
Source: Front Plant Sci. 2022 Jun 17;13:869455. doi: 10.3389/fpls.2022.869455 (PMC9247457; doi:10.3389/fpls.2022.869455)

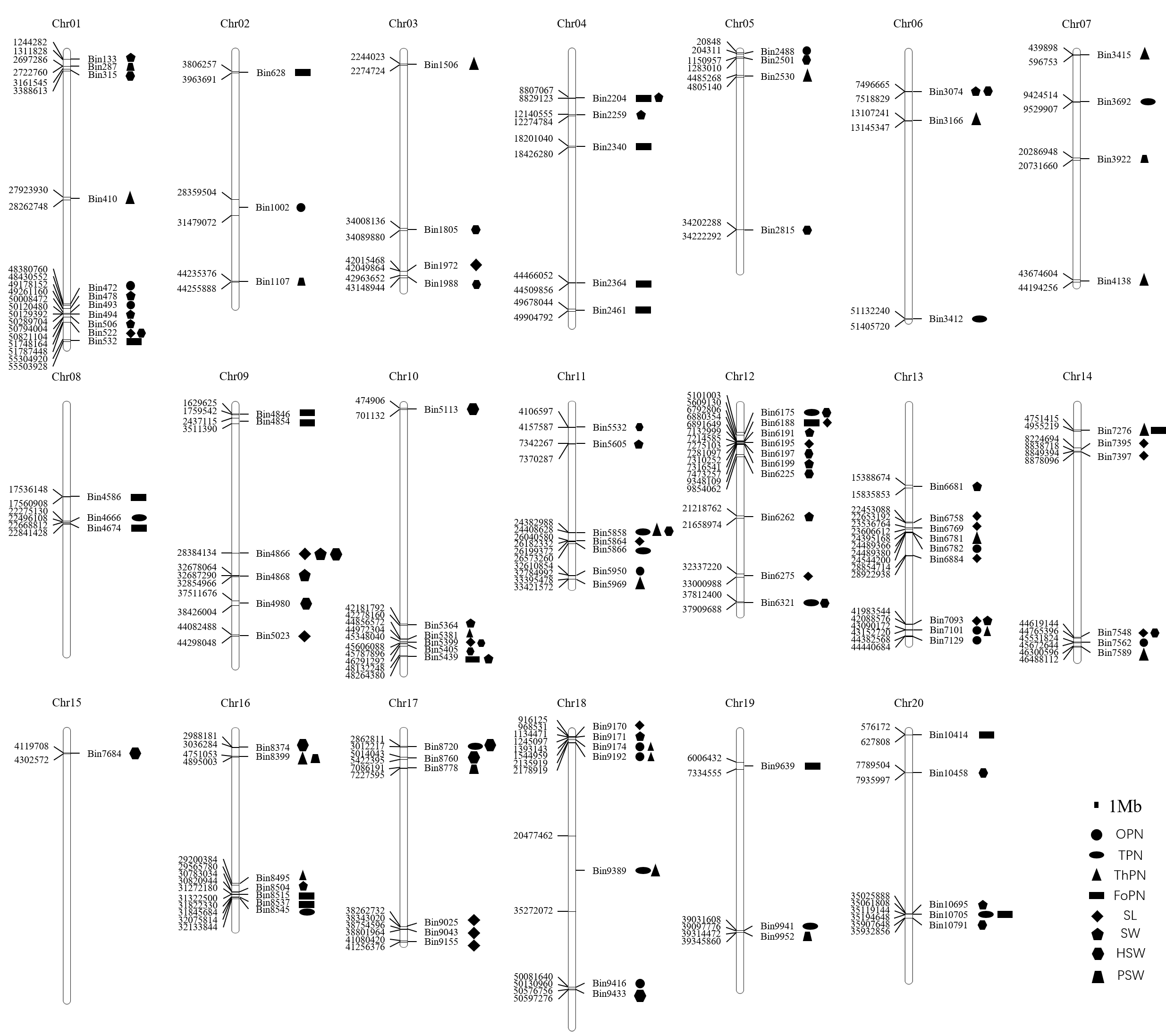

Supplement: Supplementary Figure 3 — Sequence comparison of promoter. [file Data_Sheet_3.ZIP › Supplementary figure/Supplementary figure 1.tif]

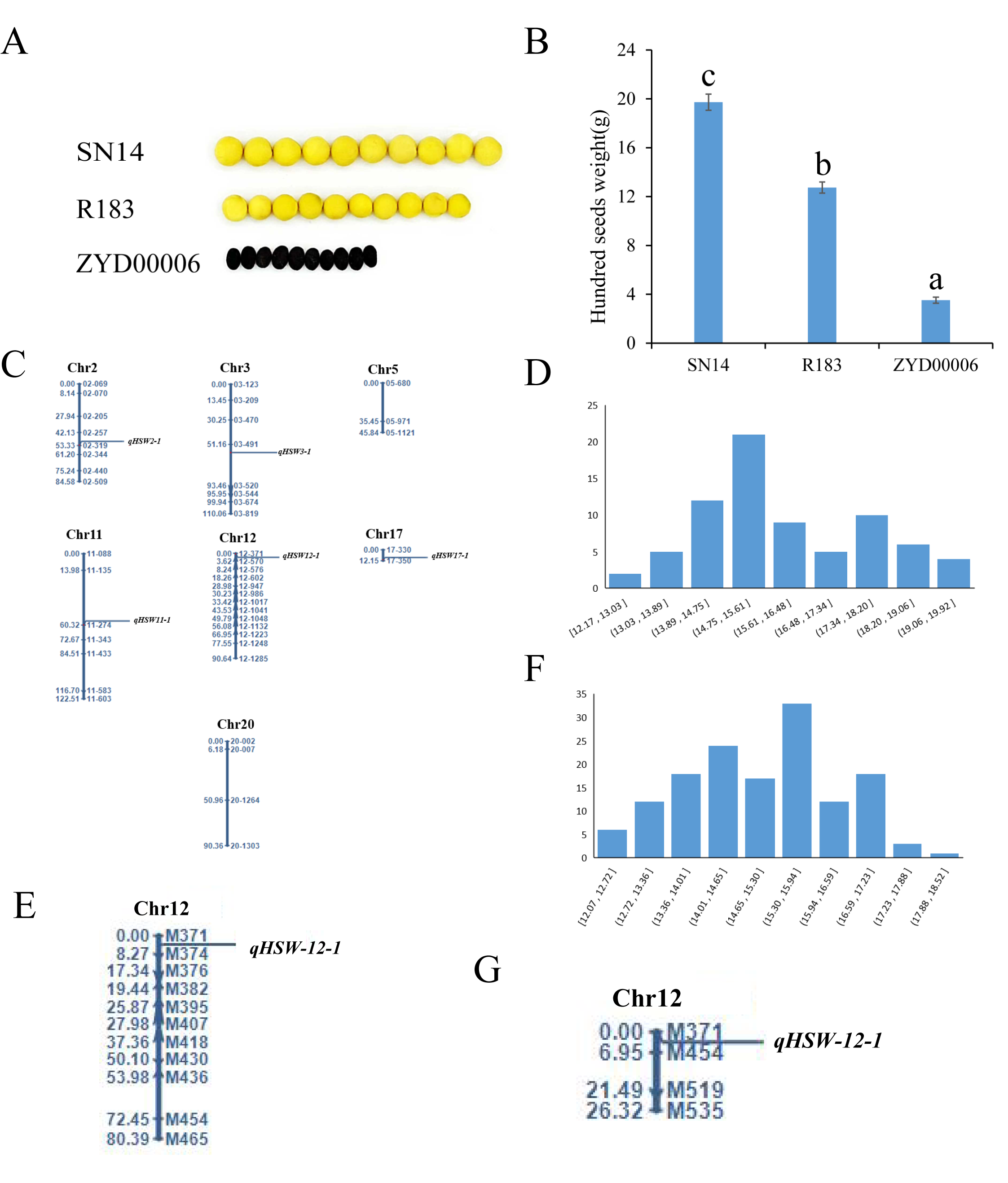

Supplement: Supplementary Figure 3 — Sequence comparison of promoter. [file Data_Sheet_3.ZIP › Supplementary figure/Supplementary figure 2.tif]
